# Validity of the Australian Recommended Food Score as a diet quality index for Pre-schoolers

**DOI:** 10.1186/1475-2891-13-87

**Published:** 2014-09-02

**Authors:** Tracy L Burrows, Kate Collins, Jane Watson, Maya Guest, May M Boggess, Melinda Neve, Megan Rollo, Kerith Duncanson, Clare E Collins

**Affiliations:** Nutrition and Dietetics, School of Health Sciences, Faculty of Health and Medicine, The University of Newcastle, >Callaghan, 2308 NSW Australia; Priority Research Centre in Physical Activity and Nutrition, Faculty of Health, The University of Newcastle, Callaghan, 2308 NSW Australia; Environmental and Occupational Health and Safety, School of Health Sciences, Faculty of Health and Medicine, The University of Newcastle, Callaghan, 2308 NSW Australia; School of Mathematical and Statistical Sciences, Arizona State University, Tempe, 85281 AZ USA; Hunter New England Local Health District, Forster, 2428 NSW Australia

**Keywords:** Diet quality index, Food frequency questionnaire, Pre-schoolers, Nutritional adequacy

## Abstract

**Background:**

Diet quality tools provide researchers with brief methods to assess the nutrient adequacy of usual dietary intake. This study describes the development and validation of a pediatric diet quality index, the Australian Recommended Food Scores for Pre-schoolers (ARFS-P), for use with children aged two to five years.

**Methods:**

The ARFS-P was derived from a 120-item food frequency questionnaire, with eight sub-scales, and was scored from zero to 73. Linear regressions were used to estimate the relationship between diet quality score and nutrient intakes, in 142 children (mean age 4 years) in rural localities in New South Wales, Australia.

**Results:**

Total ARFS-P and component scores were highly related to dietary intake of the majority of macronutrients and micronutrients including protein, β-carotene, vitamin C, vitamin A. Total ARFS-P was also positively related to total consumption of nutrient dense foods, such as fruits and vegetables, and negatively related to total consumption of discretionary choices, such as sugar sweetened drinks and packaged snacks.

**Conclusion:**

ARFS-P is a valid measure that can be used to characterise nutrient intakes for children aged two to five years. Further research could assess the utility of the ARFS-P for monitoring of usual dietary intake over time or as part of clinical management.

## Background

Diet quality refers to both nutrient adequacy and food variety within healthful food groups, as well as alignment of overall eating patterns with National Dietary Guidelines. Diet quality scores or indices are used to summarize dietary intake into a single numeric variable, which addresses some of the limitations in evaluations of diet-disease relationships based only on single nutrients [1]. However, given the major concerns related to these limitations, it is advisable to keep in mind that findings from food frequency questionnaire based epidemiological studies of diet-disease relationships could have their own limitations. In adults, chronic disease risk factors, including elevated systolic blood pressure, obesity, hyperglycemia, and both total and disease specific morbidity and mortality, are greater in those with poorer diet quality [2, 3].

The validation and reproducibility of diet quality indices in relation to health outcomes in pediatric populations is more challenging to assess for two reasons: first, the time lag to disease development [4], and second, there are fewer indices that have been validated in pediatric populations [5]. Intermediate clinical markers that have been examined previously include BMI [6, 7], percentage body fat and waist circumference [6, 8], blood pressure [6], micronutrient intakes [9], plasma lipids, inflammation markers, serum iron, vitamin B_12_ and homocysteine [10, 11]. Reviews that examine the relationship between diet quality and health outcomes in children have demonstrated modest associations with asthma and dental caries [6].

To date, only a limited number of studies have sought to validate diet quality indices against nutrient intakes in pediatric populations [12, 13]. Huybrechts et al. [12] developed a Diet Quality Index (DQI) for pre-school children and evaluated it for validity (n = 510) and reproducibility (n = 58). The DQI was shown to positively correlate with a range of macronutrients and micronutrients, and additionally, food frequency questionnaire (FFQ)-based DQI calculations showed moderate agreement with the DQI calculated from a 3 day food record [12].

The authors previously evaluated the performance of the Australian Child and Adolescent Recommended Food Score (ACARFS) compared to nutrient intakes derived from an FFQ in a population (n = 691) of children aged 9–12 years [13]. Agreement between ACARFS and nutrient intakes was demonstrated through positive correlations between ACARFS and all vitamins, minerals and total energy intake. However, that evaluation did not include younger children.

Therefore, the aims of this study were to develop a pediatric DQI, the Australian Recommended Food Score for Pre-schoolers (ARFS-P), and evaluate its performance as a measure of diet quality by assessing agreement in pre-schoolers with nutrient intakes derived from a previously validated FFQ [4, 14].

## Methods

The current study was a cross-sectional evaluation of dietary intake in young children aged 2–5 years (n = 146). The data was baseline measures of a randomized control trial, Feeding Healthy Food to Kids (FHFK), conducted in five rural low socioeconomic localities in New South Wales (NSW), Australia, in August 2009 [15, 16]. Briefly, parents of young children were recruited from childcare facilities by early childhood health professionals. Inclusion criteria were parents were aged 18 years or older and the child was aged 2–5 years. If more than one child in the family met this criterion, the eldest child within the eligible age range was selected as the study child for consistency and simplicity. Demographic variables collected included child age, gender, Aboriginal or Torres Strait Islander status, child health status, and parental education level. Four children had missing age, leaving a final sample size of n = 142. Written informed consent was obtained from all participants’ parents prior to their enrolment in the FHFK study. Approval was obtained from Hunter New England (HNE) Human Research Ethics Committee (reference number HREC/08/HNE/403) and the University of Newcastle Human Research Ethics Committee (approval number H-2009-0106).

### Assessment of dietary intake AES-P FFQ

Dietary intake was assessed using the Australian Eating Survey Pre-schooler Version (AES-P). Given the age of the pre-schoolers, a caregiver (i.e. parent or guardian) recorded the child’s frequency of consumption of a comprehensive 120-item semi-quantitative FFQ. Further details of the development of the AES FFQ have been published elsewhere [4, 14], demonstrating acceptable accuracy for ranking nutrient intakes in children and adolescents 9–16 years of age [14]. The AES-P was previously shown to be a valid estimate of total energy expenditure in children 3 years of age [4], and children 8–11 years of age using doubly labeled water [17].

Caregivers recorded their child’s frequency of consumption of a comprehensive, defined list of foods over the previous 6 months. The frequency options ranged from ‘never’ to ‘4 or more times per day’, to ‘7 or more glasses per day’ for beverages. Questions from the FFQ were grouped categorically into food groups and subgroups including those energy dense nutrient poor foods now referred to as ‘ discretionary choices’ [18]. Due to the seasonal availability of some fruits, a separate section was included in the FFQ for seasonal fruit. The frequency categories were listed as for other food items, with the question, “when the following fruit is in season, how often do you usually eat it?” to capture the usual consumption of the fruit when it is in season. Seasonal availability was determined by contacting the food markets, Sydney, NSW and obtaining information about the wider availability in other markets and supermarkets during the year, in addition to referring to supermarket literature that indicated the months of the year different seasonal fruit was available.

Pre-school age portion sizes in grams for individual foods items, was derived from the 2007 National Children’s Nutrition and Physical Activity Survey, purchased from the Australian Social Science Data Archive at the Australian National University [19]. Nutrient intakes from the AES-P FFQ were computed from the most current food composition database of Australian foods available, the Australian AusNut 2007 database (All Foods) Revision 17 and AusFoods (Brands) Revision 5 [20] to generate individual mean daily macro-and micronutrient intakes. The estimated daily intakes for 20 macro- and micronutrients were calculated using FoodWorks [21] and compared to age-specific nutrient targets. Adequacy of nutrient intake was assessed using Recommended Daily Intake (RDI) and Adequate Intake (AI) targets [22], while intakes exceeding recommendations were defined by Upper Limits (UL) [22] and the 2013 Australian Dietary Guidelines [18].

### The Australian Recommended Food Score for Pre-Scoolers ARFS-P

The ARFS-P was designed as a brief, culture specific, food based diet quality tool for young children, focusing on dietary variety within recommended food groups. It was modeled on the Recommended Food Score [23] and the Australian Recommended Food Score (ARFS) [24], and utilizes a subsample of questions from the AES-P FFQ consistent with the 2013 Australian Dietary Guidelines [18]. The ARFS-P includes seventy questions relating to eight food group components; vegetables (n = 20), fruit (n = 12), meat (n = 7), meat alternatives (i.e. non-meat protein) (n = 6), breads/cereals (n = 12), dairy (n = 10), water (n = 1) and condiments (n = 2). The procedure used to calculate ARFS has been published elsewhere [13]. Briefly, points were awarded for foods consumed according to frequency of consumption, with healthy foods receiving more points (i.e. vegetables, low-fat dairy, whole grains), and point caps on foods that should not be consumed overly often (e.g. meat, whole milk). An ARFS component was only calculated for those responses with not more than one question missing; one child was excluded for this reason. The ARFS total possible score ranges from zero to 73, and the component scores from zero to the number of questions in that component, plus one more possible for vegetables, low-fat dairy, whole grains.

### Statistical analysis

Scores were described by medians and interquartile range (IQR) and Fisher’s exact test and Wilcoxon rank-sum test were used to compare two age groups in univariate analyses. Groups were younger children (<4 years of age) and older children (≥4 years of age), so that the same RDI, AI and UL values would apply to all participants in a group. Linear regressions were used to estimate the beta-coefficients (which can be thought of as correlations) of ARFS-P scores with FFQ components, whilst controlling for demographic factors. More precisely, agreement was assessed using linear regression models with AES-P FFQ food group, macro and micronutrient intakes as response variables and ARFS-P components as explanatory variables. Demographic variables, age, gender and parent education, were controlled for by being kept in the model if significant (too few participants were Aboriginal or Torres Strait Islander to be able to estimate the effect of race). The total energy intake was included as an explanatory variable if significant. By this we mean that backwards stepwise with p = 0.05 for removal was employed to remove variables that added no predictive value to the model. This was followed by adding any variable back in that was significant at the 0.05 level, so that that smallest model with the best predictive ability was identified. Both ARFS-P and FFQ were standardized (subtract mean and divide by standard deviation) so the model coefficient was the beta-coefficient. A square root transform was applied to some variables to improve the normality of the residuals. Kolmogorov-Smirnov tests and normal probability plots were used to assess normality of residuals. Square root transform of response variables was used if necessary to improve residual normality. Statistical significance was at the 5% level. All data manipulation and statistical analysis was undertaken using Stata MP v12 [25].

## Results

Table 1 lists demographic characteristics of participants (n = 142) by age group. No significant difference across age groups was found for gender, chronic health conditions or race.

**Table 1:** **Demographic characteristics of study population of pre-schoolers, by age group**

	All	Younger < 4 yrs	Older ≥ 4 yrs	P
	n = 142	n = 76	n = 66	
**Gender**				
*Female*	66 (46%)	40 (53%)	26 (39%)	
*Male*	76 (54%)	36 (47%)	40 (61%)	0.13
**Chronic Health Condition**				
*Yes*	4 (3%)	3 (4%)	1 (2%)	
*No*	138 (97%)	73 (96%)	65 (98%)	0.62
**Aboriginal/ Torres Strait Islander**				
*Yes*	5 (4%)	3 (4%)	2 (3%)	
*No*	137 (96%)	73 (96%)	64 (97%)	1.00
**Parent Education**				
*Did not complete Year 10*	3 (2%)	1 (1%)	2 (3%)	
*Completed year 10*	37 (26%)	16 (21%)	21 (32%)	
*Completed Year 12*	25 (18%)	19 (25%)	6 (9%)	
*Commenced Higher Education Degree*	14 (10%)	6 (8%)	8 (12%)	
*Completed Higher Education Degree*	42 (30%)	20 (26%)	22 (33%)	
*Completed Post Graduate Course*	21 (15%)	14 (18%)	7 (11%)	0.07

Table 2 reports the percentage of participants that met age specific nutrient RDIs and AIs, and the percentage that exceeded UL for sodium and the percentage energy (%E) from saturated fat, according to their AES-P FFQ reported food intake. Over 75% of participants met recommendations for the majority of macronutrients and micronutrients with the exception of fibre, iron and potassium. In fact, less than 20% met the daily requirement for iron. Only five percent of participants met recommendations in the Dietary Guidelines in Australia of less than 10% of total energy derived from saturated fat, while 81% of younger children and 61% of older children exceeded the recommended upper limit for sodium intake.

**Table 2:** **Comparison of nutrient intakes, as assessed by the Australian Eating Survey Pre-schooler Version (AES-P) Food Frequency Questionnaire, to Australian Recommended Dietary Intakes (RDI), Adequate Intake (AI) and upper limit (UL), by age group**

Intake per day	Younger < 4 yrs (n = 76)			Older ≥ 4 yrs (n = 66)		
Meeting ^1^	RDI/AI	Mean	Meeting	RDI/AI	Mean	Meeting
*Protein (g)*	14	57.23	100%	20	53.13	100%
*Fibre (g)*	14	16.13	62%	18	15.9	44%
*Vitamin A (μg)*	300	784.39	99%	400	735.54	97%
*Thiamine (mg)*	0.5	1.4	99%	0.6	1.37	99%
*Riboflavin (mg)*	0.5	2.27	100%	0.6	2.16	100%
*Niacine equiv (mg)*	6	25.52	100%	8	24.26	100%
*Folate (μg)*	150	223.91	89%	200	222.95	77%
*Vitamin C (mg)*	35	84.14	97%	35	79.92	96%
*Calcium (mg)*	500	1014.41	96%	700	945.23	87%
*Iron (mg)*	9	7.84	22%	10	7.97	18%
*Magnesium (mg)*	80	246.59	100%	130	239.72	100%
*Phosphorus(mg)*	460	1151.03	100%	500	1073.8	100%
*Potassium(mg)*	2000	2379.39	68%	2300	2188.1	54%
*Zinc (mg)*	3	7.72	100%	4	7.3	99%
**Exceeding**	**UL**	**Mean**	**Exceeding**	**UL**	**Mean**	**Exceeding**
*Sodium(mg)* ^1^	1000	1329.39	82%	1400	1342.27	61%
*%E Saturated fat* ^2^	10	16.83	95%	10	15.95	95%

^1^Nutrient Reference Values for Australia and New Zealand [22].

^2^Australian Dietary Guidelines [18].

Table 3 reports the ARFS-P score, component scores, and macronutrient intake and micronutrient intake descriptive statistics, by age group. The median ARFS-P score was 36 with a minimum of 12 and a maximum of 55, with no significant difference by age. There were no significant differences by age in any component ARFS-P, or macro or micronutrients. Marginal significance (p < 0.1) was reached with saturated fat and cholesterol. Also in Table 3 is the %E from nutrient dense and discretionary choice components, as assessed by the AES-P FFQ, by age group. The median %E from nutrient dense food was 70.5%, and 29.5% from discretionary choices, with no significant difference by age. However, older children did obtain significantly higher %E from grains and sweetened cereals than their younger counterparts, and marginally so for packaged snacks.

**Table 3:** The Australian Recommended Food Scores for Pre-schoolers (ARFS-P) and Australian Eating Survey Pre-schooler Version (AES-P) Food Frequency Questionnaire (FFQ) nutrients, and % energy from nutrient-dense and discretionary choices, by age (*includes one for adequate water intake)

	All (n = 142)		Younger < 4 yrs (n = 76)		Older ≥ 4 yrs (n = 66)		
ARFS-P (total possible score)	Median	IQR	Median	IQR	Median	IQR	P
Total (73)*	36	(29–42)	35	(29–42)	36	(29.5–41.5)	0.97
*Vegetables (21)*	11	(8–15)	11	(8–16)	11.5	(9–14)	0.91
*Fruit (12)*	8	(6–10)	8	(6–10)	8	(5.5–9.5)	0.67
*Meat (7)*	2	(2–3)	2	(2–3)	2	(2–3)	0.84
*Meat alternatives (6)*	2	(1–2)	1	(1–2)	2	(1–3)	0.78
*Grains (13)*	6	(4–7)	6	(4–7)	6	(4–7)	0.62
*Dairy (11)*	5	(4–6)	5	(4–6)	5	(4–6)	0.84
*Condiments (2)*	1	(1–2)	1	(1–2)	2	(1–2)	0.31
**AES-P FFQ macronutrients**							
*Energy (kJ)*	5216	(4647–6242)	5226	(4656–6379)	5182	(4577–5944)	0.43
*Protein (g)*	53.79	(47–63)	54.6	(48.0–63.9)	52.58	(44.7–60.1)	0.14
*Saturated fat (g)*	23.25	(17–28)	24.44	(19.6–28.7)	21.18	(16.5–26.9)	0.06
*Cholesterol (mg)*	161.7	(126–206)	167.3	(137–209)	155.9	(121–200)	0.09
*Carbohydrate (g)*	150.0	(130–177)	148.7	(129–181)	152.0	(133–175)	0.93
*Sugars (g)*	88.09	(72.9–109)	87.79	(73.7–110)	88.2	(69.9–106)	0.73
*Fibre (g)*	15.33	(12.4–18.0)	15.27	(11.7–18.6)	15.37	(12.9–17.8)	0.91
**AES-P FFQ micronutrients**							
*Vitamin A (μg)*	745.0	(604–891)	768.5	(620–914)	715.8	(594–878)	0.25
*Retinol (μg)*	339.5	(225–439)	359.2	(238–482)	314.1	(210–415)	0.08
*β-carotine (μg)*	2497	(1773–3049)	2580	(1676–3145)	2353	(184–2818)	0.65
*Thiamine (mg)*	1.28	(1.0–1.6)	1.28	(1.0–1.7)	1.28	(1.0–1.5)	0.96
*Riboflavin (mg)*	2.12	(1.7–2.6)	2.29	(1.7–2.6)	2.11	(1.6–2.5)	0.38
*Niacine equivalent (mg)*	23.75	(20.9–27.4)	24.01	(20.9–28.5)	23.71	(20.8–27.1)	0.60
*Folate (μg)*	211.2	(182–255)	211.2	(176–261)	210.2	(184–241)	0.95
*Vitamin C (mg)*	78.12	(59.9–100)	78.12	(63.2–102)	78.69	(59.6–98.3)	0.73
*Calcium (mg)*	963.2	(746–1167)	988.9	(828–1179)	889.9	(711–1093)	0.10
*Iron (mg)*	7.72	(6.1–8.9)	7.51	(6.1–8.9)	7.91	(6.2–8.7)	0.62
*Magnesium (mg)*	227.5	(202–277)	227.5	(202–278)	227.5	(202–275)	0.69
*Potassium (mg)*	2208	(1897–2618)	2311	(1931–2700)	2132	(1869–2494)	0.14
*Phosphorus (mg)*	1109	(900–1292)	1121	(956–1304)	1073	(869–1220)	0.15
*Sodium (mg)*	1279	(1052–1594)	1279	(1029–1603)	1287	(1097–1559)	0.69
*Zinc (mg)*	7.36	(6.3–8.5)	7.67	(6.4–8.7)	7.14	(6.2–8.1)	0.16
**% Energy from**							
*Saturated Fat*	17	(14.0–19.0)	17	(14.0–19.0)	16	(13.5–18.0)	0.12
***Nutrient dense***	71	(63–79)	72	(64–81)	70.5	(62.5–79)	0.29
*Vegetables*	5	(3–7)	5	(3–7)	5	(4–7)	0.78
*Fruit*	10	(7–15)	12	(8–16)	10	(7–13.5)	0.30
*Meat*	7	(5–9)	7	(5–9)	7	(5–9.5)	0.77
*Meat alternatives*	1	(1–3)	1	(1–3)	1	(1–3)	0.74
*Grains*	18	(14–21)	16	(13–20)	19	(15–22)	0.03
*Dairy*	28	(22–35)	29	(22–36)	26.5	(21.5–32.5)	0.10
**Discretionary choices**	29	(21–37)	28	(19–36)	29.5	(21–37.5)	0.29
*Sweetened drinks*	2	(1–4)	2	(0–4)	2.5	(1–5)	0.19
*Packaged snacks*	4	(1–7)	3	(1–6)	4	(2–7)	0.09
*Confectionary*	3	(2–5)	4	(2–5)	3	(2–5)	0.81
*Baked sweet products*	5	(3–8)	5	(3–7)	4.5	(3–8)	0.92
*Take-away*	5	(3–6)	5	(3–7)	4	(3–6)	0.59
*Condiments*	2	(1–4)	2	(1–3)	3	(1–4)	0.14
*Processed Meats*	1	(1–2)	1	(1–2)	1	(1–2.5)	0.51
*Sweet breakfast cereal*	8	(5–11)	7	(5–10)	9	(7–11.5)	0.01

### Assessment of Agreement between AFRS-P and AES-P FFQ nutrients and food groups

Table 4 demonstrates that total ARFS-P and the ARFS-P components were significantly related to AES-P FFQ intake of the majority of macro and micronutrients. Total ARFS-P was positively related to protein (ρ = 22% CI_95%_ 14-30), cholesterol (ρ = 27% CI_95%_ 15-40), fibre (ρ = 12% CI_95%_ 3-22), vitamin A (ρ = 40% CI_95%_ 26-54), β-carotine (ρ = 58% CI_95%_ 44-71), niacine equivalents (ρ = 20% CI_95%_ 10-30), folate (ρ = 14% CI_95%_ 2-26), vitamin C (ρ = 44% CI_95%_ 31-58), calcium (ρ = 16% CI_95%_ 4-29), magnesium (ρ = 16% CI_95%_ 9-24), phosphorus (ρ = 13% CI_95%_ 6-21), potassium (ρ = 27% CI_95%_ 19-35) and zinc intake (ρ = 17% CI_95%_ 10-25), while negatively related to carbohydrate (ρ = -10% CI_95%_ -16- -5).

**Table 4:** **Beta-coefficients relating intakes as assessed by the Australian Eating Survey Pre-schooler Version (AES-P) Food Frequency Questionnaire (FFQ), and the Australian Recommended Food Score for Pre-schoolers (ARFS-P) components of standardized ARFS component in linear regression model of standardized FFQ nutrient, adjusted for total FFQ energy and demographics age, gender and education (n = 142)**

	ARFS-P Total	ARFS-P Veg	ARFS-P Fruit	ARFS-P Meat	ARFS-P Altern.	ARFS-P Grains	ARFS-P Dairy	ARFS-P Condmt
**AES-P FFQ Macronutrients**								
*Protein (g)*	0.220**	0.176**	0.132**	0.263**			0.191**	
*Saturated fat (g)*								
*Cholesterol (mg)*	0.271**	0.195**	0.185**	0.382**	0.195**	0.127		-0.156*
*Carbohydrate (g)*	-0.104**	-0.100**		-0.128**		-0.052	-0.057	
*Sugars (g)*				-0.073			-0.093*	
*Fibre (g)*	0.125**	0.121*	0.173**		0.091		-0.082	
**AES-P FFQ Micronutrients**								
*Vitamin A (μg)*	0.399**	0.442**T	0.326**	0.287**				
*Retinol (μg)*				0.187*T				
*β-carotine (μg)*	0.578**X	0.622**X	0.469**X	0.246**X		0.235**X		
*Thiamine (mg)*								0.246**T
*Riboflavin (mg)*			0.133					0.199**
*Niacine equivalent (mg)*	0.171**T	0.123*	0.138**	0.152**			0.158**	0.147**
*Folate (μg)*	0.141*	0.138*	0.165**					0.105
*Vitamin C (mg)*	0.441**	0.444**	0.517**	0.251**				
*Calcium (mg)*	0.164*	0.145*	0.111	0.125E			0.156*	
*Iron (mg)*								
*Magnesium (mg)*	0.164**	0.126**	0.133**			0.083*		
*Potassium (mg)*	0.273**M	0.271**M	0.281**M	0.163**E				
*Phosphorus (mg)*	0.132**	0.110**	0.068E	0.130**E			0.149**	
*Sodium (mg)*			-0.103*	-0.083			0.194**	0.176**
*Zinc (mg)*	0.173**	0.174**	0.137**	0.154**			0.092*	
**% Energy from**								
*Saturated Fat*							-0.149X	
**Nutrient dense Foods**	0.485**	0.476**	0.511**	0.355**	0.178*	0.231**		-0.170*
*Vegetables*	0.518**	0.637**	0.348**	0.232**	0.170*	0.208**		
*Fruit*	0.306**X	0.277**X	0.537**				-0.207*X	
*Meat*	0.276**X	0.244**X	0.214*X	0.535**				
*Meat alternatives*	0.316**T	0.198*T	0.170*T		0.673**	0.279**T		
*Grains*				-0.171*X				
*Dairy*								
**Discretionary Choices**	-0.484**	-0.476**	-0.509**	-0.355**	-0.177*	-0.231**		0.171*
*Sugar sweetened drinks*	-0.333**T	-0.272**	-0.302**T			-0.192*T		
*Packaged snacks*	-0.379**	-0.284**	-0.409**	-0.187*T	-0.249**T	-0.271**T		
*Confectionary*	-0.199*X	-0.223**X		-0.209*X		-0.152X		
*Baked sweet products*			-0.149X	-0.162X				
*Take-away*	-0.354**X	-0.328**X	-0.290**X	-0.260**X	-0.199*X	-0.188*X		
*Condiments*		-0.256**X			0.258**X			
*Processed Meats*	-0.195*X	-0.199*X		-0.159X	-0.178*X	-0.203*X		
*Sweet breakfast cereal*				-0.143X	-0.172*X		-0.147X	

Only beta-coefficients with P < 0.10 are shown; *5% significance, **1% significance.

(T) square root transform was applied, (M) gender significant in model, (E) parent education significant in model, (X) energy not significant in model.

Shaded values indicate a negative relationship.

**Figure 1:**
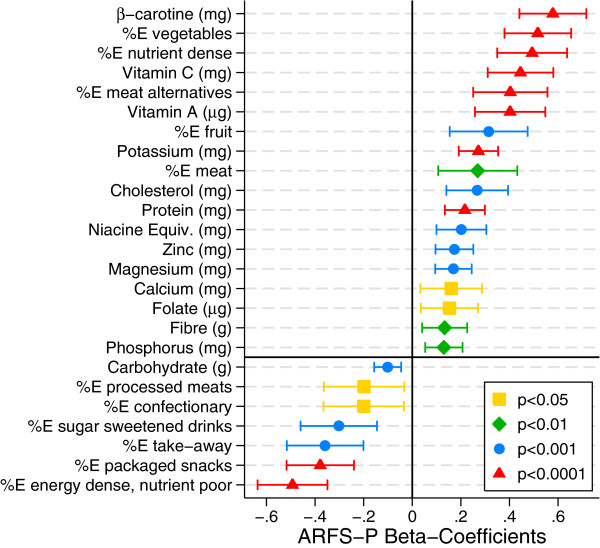
**Significant beta-coefficients of ARFS-P component of AES-P nutrients and%E from food groups, controlling for total energy consumption, age, gender and parent education, as estimated by linear regression modelling, with 95% confidence intervals.**

Table 4 also demonstrates that total ARFS-P was significantly related to eating patterns. It was positively related to total consumption of nutrient-dense foods, as well as food sub-groups including vegetables, fruit, meat and meat alternatives. In contrast, total ARFS-P was negatively related to total consumption of discretionary choices, and subgroups such as confectionary, sugar sweetened drinks, packaged snacks and take-away foods. Figure 1 displays these beta-coefficients between total ARFS-P and AES-P FFQ reported intake, together with their 95% confidence intervals.

## Discussion

The importance of consuming a large variety of nutritious foods is recognized within national food guidelines [18]. The ARFS-P was developed to reflect the food behaviors and eating habits recommended in such guidelines. The use of brief tools is convenient for clinicians and allows for rapid individualized feedback and early interventions targeting improvements in diet quality. This is important given recent reviews of diet quality indices in children suggesting healthier diet patterns are associated with cognition, behavior, and anthropometric factors [6, 7]. Associations between diet quality indices and short and long term health outcomes, such as blood pressure, asthma, blood lipids, inflammation factors and dental caries, has also been recognized [6].

This study is novel in applying this approach to assess diet quality in a pre-school population. We have previously validated this diet quality scoring method, which uses a food-based approach in an older pediatric population aged 9–16 years [13]. While diet quality scores have been constructed for use in children, recent reviews have highlighted the need for additional studies in more diverse population samples with analyses adjusted more fully for potential confounders [6, 7] and a specific need for further studies in children under the age of five years [6, 7, 26]. The ARFS-P was calculated from intakes of whole foods rather than nutrient intakes, making this method of interest when information about usual food intake, as opposed to nutrients, is sought or when feedback to individuals about their food patterns would be useful.

While few DQIs have been developed specifically for use with toddler populations [27], findings from this study are similar to other diet quality validation studies in toddlers, including the FFQ DQI developed for pre-schoolers in which significant correlations were found between their DQI and nutrients such as protein, fibre, calcium and zinc [12], as we did in this study (r = 0.220, p < 0.001; r = 0.125, p = 0.008; r = 0.164, p = 0.011; r = 0.173, p < 0.001; respectively). However, we found a strong relationship between ARFS-P and β-carotine where they did not; differences such as these are not surprising since they used Pearson's correlations that do not allow for controlling for influences of, for instance total energy and age. Other validation studies of diet quality scores, such as the Healthy Eating index [28], found strong relationships with dietary intake, including fibre, folate and vitamin C, as did we (r = 0.125, p = 0.008; r = 0.141, p = 0.018; r = 0.441, p < 0.001; respectively).

Strategies to enhance diet quality need to focus on increasing the variety of nutrient-dense foods in a child’s diet, and reducing consumption of discretionary choices. Results of the current study indicate that each of the ARFS component scores were low compared to the total maximum point available, particularly for sub-scales of fruit and vegetables. This suggests that increasing the variety of fruit and vegetables consumed regularly could be important areas to target improvements in diet quality. Other areas, in descending order, could include lean meat and vegetarian protein alternatives. This suggests that public health messages targeting increases in the variety amongst healthful foods that are consumed each week, could offer a new approach to nutrition promotion.

This study found that ARFS-P scores were highly related to both nutrient intakes and consumption patterns of children aged two to five years. The sensitivity of this DQS is demonstrated by its ability to detect significant changes in macronutrient and micronutrient intake per unit change in ARFS-P. By way of example, the positive beta-coefficient of β-carotene with total ARFS-P means that an increase in total ARFS-P corresponds to an increase in pre-schooler β-carotene intake, on average. A similar trend was evident when considering intake of food groups and ARFS-P. Consequently the use of the ARFS-P was valid in assessing the eating patterns and nutrient intakes of the study population. Marginal significance was reached with saturated fat and cholesterol, providing some evidence that younger children may have been consuming more saturated fat and cholesterol than their older counterparts.

### Strengths and limitations

Anthropometric data, such as weight, were not obtained from this population, since the primary outcome measure of the FHFK randomized controlled trial was changes to dietary intake. As a consequence, the relationship between weight status and ARFS-P could not be explored. However, unpublished data collected in 2008 as part of the Before School Screening program for 4–5 year olds (n = 571) in the same study locations suggested that rates of overweight and obesity were higher than the NSW average [29], with 26.8% of children overweight or obese (27.3% male, 26.3% female). Despite a previous study suggesting that the FFQ was relatively accurate when assessing total energy intake at the group level [4], over –and under-reporting of dietary intake and physical activity level were not assessed in the current study. The relatively small sample from rural locations reduces the generalizability and hence results should be interpreted with caution.

The effect of parental bias must also be considered when interpreting results as a caregiver completed the AES-P on their child’s behalf. This may exacerbate the over reporting bias already associated with the use of an FFQ [30] and may increase the chance that the data is incomplete or that intake has been underestimated [31]. Future studies should consider obtaining dietary intake reports from both parents, or where appropriate, with the child present to try to reduce reporting biases [32, 33].

Some limitations of calculating and using ARFS to determine usual diet quality have been previously published [13], including that the national nutrient databases used in this study does not accurately reflect population consumption of folate as fortification of breads, cereals and cereal products is not accounted for within the database, hence results obtained for FFQ folate intake are likely to be lower than true intake.

### Implications for research and practice

The ARFS-P allows for the rapid measurement of diet quality of children aged 2–5 years in a single continuous variable. It was derived from a validated FFQ for young children, and provides researchers the opportunity to use an independent measure of intake, or calculated secondarily, from the AES-P FFQ. Its calculation is less onerous than methods that require derivation of nutrient based sub-scales. This means that the provision of feedback based on overall score can potentially be in real-time.

Based on the finding in the current study, the ARFS-P method for scoring diet quality could be applied to other FFQs, in other populations, age groups, and in other settings. This could include testing its use as a self-monitoring tool or within clinical practice. If validated in these settings it would be a convenient tool that could be used by a variety of health professionals to assess and monitor dietary patterns.

## Conclusions

In conclusion, the ARFS-P is a brief dietary index that assesses usual diet quality, food variety and the nutritional adequacy of dietary intakes of Australian pre-schoolers. It was a valid tool that can be used to characterize nutrient intake as well as eating patterns. Future research should examine whether the ARFS-P can be used to target improvements in diet quality within interventions and/or population health approaches aimed at optimizing the dietary patterns of young children.

## Abbreviations

DQI: Diet Quality Index
FFQ: Food Frequency Questionnaire
ACARFS: Australian Child and Adolescent Recommended Food Score
ARFS: Australian Adolescent Recommended Food Score
ARFS-P: Australian Adolescent Recommended Food Score for Pre-schoolers
HNE: Hunter New England
FHFK: Feed Healthy Food to Kids
NSW: New South Wales
AES: Australian Healthy Eating Survey
AES-P: Australian Healthy Eating Survey Pre-schooler version
RDI: Recommended Daily Intake
AI: Adequate Intake
UL: Upper Limits
%E: Percent energy.
